# rA1M-035, a Physicochemically Improved Human Recombinant α_1_-Microglobulin, Has Therapeutic Effects in Rhabdomyolysis-Induced Acute Kidney Injury

**DOI:** 10.1089/ars.2017.7181

**Published:** 2018-12-27

**Authors:** Bo Åkerström, Lena Rosenlöf, Anneli Hägerwall, Sigurbjörg Rutardottir, Jonas Ahlstedt, Maria E. Johansson, Lena Erlandsson, Maria Allhorn, Magnus Gram

**Affiliations:** ^1^ Sections for Infection Medicine, Department of Clinical Sciences, Lund University, Lund, Sweden.; ^3^ Sections for Obstetrics and Gynecology, Department of Clinical Sciences, Lund University, Lund, Sweden.; ^2^ A1M Pharma AB, Lund, Sweden.

**Keywords:** alpha-1-microglobulin, acute kidney injury, antioxidant, reductase, radical scavenger, heme-binding

## Abstract

***Aims:*** Human α_1_-microglobulin (A1M) is an endogenous reductase and radical- and heme-binding protein with physiological antioxidant protective functions. Recombinant human A1M (rA1M) has been shown to have therapeutic properties in animal models of preeclampsia, a pregnancy disease associated with oxidative stress. Recombinant A1M, however, lacks glycosylation, and shows lower solubility and stability than A1M purified from human plasma. The aims of this work were to (i) use site-directed mutagenesis to improve the physicochemical properties of rA1M, (ii) demonstrate that the physicochemically improved rA1M displays full *in vitro* cell protective effects as recombinant wild-type A1M (rA1M-wt), and (iii) show its therapeutic potential *in vivo* against acute kidney injury (AKI), another disease associated with oxidative stress.

***Results:*** A novel recombinant A1M-variant (rA1M-035) with three amino acid substitutions was constructed, successfully expressed, and purified. rA1M-035 had improved solubility and stability compared with rA1M-wt, and showed intact *in vitro* heme-binding, reductase, antioxidation, and cell protective activities. Both rA1M-035 and rA1M-wt showed, for the first time, potential *in vivo* protective effects on kidneys using a mouse rhabdomyolysis glycerol injection model of AKI.

***Innovation:*** A novel recombinant A1M-variant, rA1M-035, was engineered. This protein showed improved solubility and stability compared with rA1M-wt, full *in vitro* functional activity, and potential protection against AKI in an *in vivo* rhabdomyolysis mouse model.

***Conclusion:*** The new rA1M-035 is a better drug candidate than rA1M-wt for treatment of AKI and preeclampsia in human patients.

Innovation α_1_-microglobulin (A1M) is a reductase and radical- and heme-binding protein with physiological antioxidant protective functions. Using three amino acid substitutions, a new recombinant human A1M (rA1M)-variant (rA1M-035) was produced, with better solubility and stability than rA1M-wt, having intact functional protective properties and protective effects. Both rA1M-wt and the new rA1M-035-variant displayed *in vitro* and *in vivo* properties, suggesting that the protein may be used for treatment of acute kidney injury (AKI). The superior stability and solubility of rA1M-035 show that this new variant is a better drug candidate than rA1M-wt.

## Introduction

Oxidative stress is a collective term used to describe conditions with an abnormally high production of oxidative and reductive compounds and/or impaired antioxidative tissue defence systems (21). Mediators of oxidative stress are reactive oxygen species (ROS), including free radicals, which are highly reactive due to the presence of unpaired electrons. ROS and radicals react with proteins, DNA, membranes, and so on, leading to unwanted modifications of the target molecules and loss of their functions. Heme-carrying proteins, such as hemoglobin (Hb), myoglobin, and cytochromes, are important contributors of oxidative stress when released from their cellular encapsulation, mainly because redox reactions between the iron of the heme-group and oxygen generate ROS (14).

Several physiological antioxidation defence systems have evolved to counteract the chemical threat of ROS, radicals, and free heme-proteins. The plasma and tissue protein α_1_-microglobulin (A1M) is one such physiologically occurring antioxidant. A1M is a 26 kDa plasma and tissue protein that has been identified in mammals, birds, fish, and amphibians (reviewed in Ref. (3, 4)]. It is synthesized in the liver at a high rate, secreted into the blood stream, and rapidly transported across the vessel walls to the extravascular compartment of all organs (26). A1M is found both in free form and as a complex with immunoglobulin A, albumin, and prothrombin in blood and interstitial tissues (12). Based on its crystal structure (28) as a cup-shaped barrel consisting of eight antiparallel β-strands enclosing a pocket with a closed bottom and an open end, it belongs to the Lipocalins, which is a protein family with 40–50 protein members in all branches of life (bacteria, plants, fungi, animals) (2).

Five properties of A1M form the basis of its proposed physiological protective role. First, three enzymatic activities work together to eliminate heme and free radicals. (i) A catalytic reductase activity is dependent on a free, unpaired cysteine (C34) with a reactive thiol group, located on a loop at the open end of the lipocalin pocket (8). NADH, NADPH, and ascorbate can function as electron-donating cofactors. The reductase activity is most likely dependent on a one-electron transfer mechanism, as suggested for the reactions with cytochrome c (8) and the synthetic radical 2,2′-azino-bis-(3-ethylbenzothiazoline-6-sulfonic acid) (ABTS) (5). (ii) Small organic radicals are covalently trapped to lysyl- and tyrosyl side-chains of A1M (5, 11, 40). (iii) A1M binds heme (7, 23, 25, 39, 41) and a processed form of the protein (t-A1M), generated by reactions with Hb, degrades the heme-group (7). (iv) In addition to these enzymatic properties, Hb and ROS induce an increased expression of A1M in several cell types, including liver and blood cell lines (32) and in hemoglobinemia conditions *in vivo* (10, 19, 33, 34). (v) A1M is incorporated by injured cells to the mitochondrial respiratory Complex I, preventing mitochondrial swelling and loss of ATP production during oxidative stress conditions (37).

A large-scale production system of recombinant human A1M (rA1M), based on the human A1M (denoted rA1M-wild-type [wt]) displaying these properties, was developed (24) with the intention to investigate the possibilities of treating conditions associated with oxidative stress. These studies have shown that rA1M protects cells and tissues from Hb-, heme-, and ROS-induced cell damage and we have shown therapeutic effects of rA1M in *in vitro* cell models (35, 36), an *ex vivo* organ perfusion model (27), *ex vivo* skin culture (31), and *in vivo* animal disease models (20, 30, 42, 45). However, rA1M-wt lacks glycosylation and displays poor solubility and stability compared with human A1M purified from urine or plasma. The lack of stability and solubility of the protein limits its use as a drug for human use, mainly due to difficulties to obtain highly concentrated solutions and long-term storage conditions in buffers at physiological pH and salt conditions. Therefore, by expressing in *Escherichia coli* a number of animal A1M-homologues and rA1M-variants with amino acid substitutions, and screening basic physicochemical and enzymatic properties, we have produced an altered rA1M-variant,“rA1M-035” (Rosenlöf *et al.*, article under preparation, titled: Production of a recombinant alpha-1-microglobulin-variant with improved structural properties).

Acute kidney injury (AKI) occurs frequently as a complication of serious illnesses and following medical procedures such as major surgery and solid organ transplantation (18, 22, 38). The pathogenic mechanisms of AKI are incompletely understood, but oxidative stress originating from inflammation, cell death, and hemolysis, in combination with a reduced blood flow in the kidneys, is described to be a major contributor to AKI (15). The pathology of AKI, plus the fact that a significant part of infused rA1M localizes to the kidney cortex (1, 26), suggests that rA1M can be used to prevent, ameliorate, or treat AKI, especially in forms of AKI where free heme plays an important pathogenic role. A widely used animal model of such AKI is the rhabdomyolysis model, in which the damage is caused by the release of thigh muscle heme, mainly from myoglobin, into the circulation and subsequent accumulation in different tissues. Furthermore, myolysis of the thigh muscle leads to release of cell debris (*e.g.*, cytosolic content, mitochondria, nuclear content, and membrane fractions), generation of free radicals, and induction of oxidative stress, tissue, and vascular damage (29, 43). Hemolysis and subsequent release of Hb and heme, and possibly generation of free radicals, are all factors that are believed to contribute to the observed AKI.

In this work, we have compared in-depth physicochemical, enzymatic, and *in vitro* cell protective properties of rA1M-wt and rA1M-035. We have also used a mild variant of the rhabdomyolysis-induced AKI model to compare the *in vivo* protective effects of the two rA1M variants on kidneys.

## Results

### Expression, purification, and physicochemical properties

**FIG. 1.**
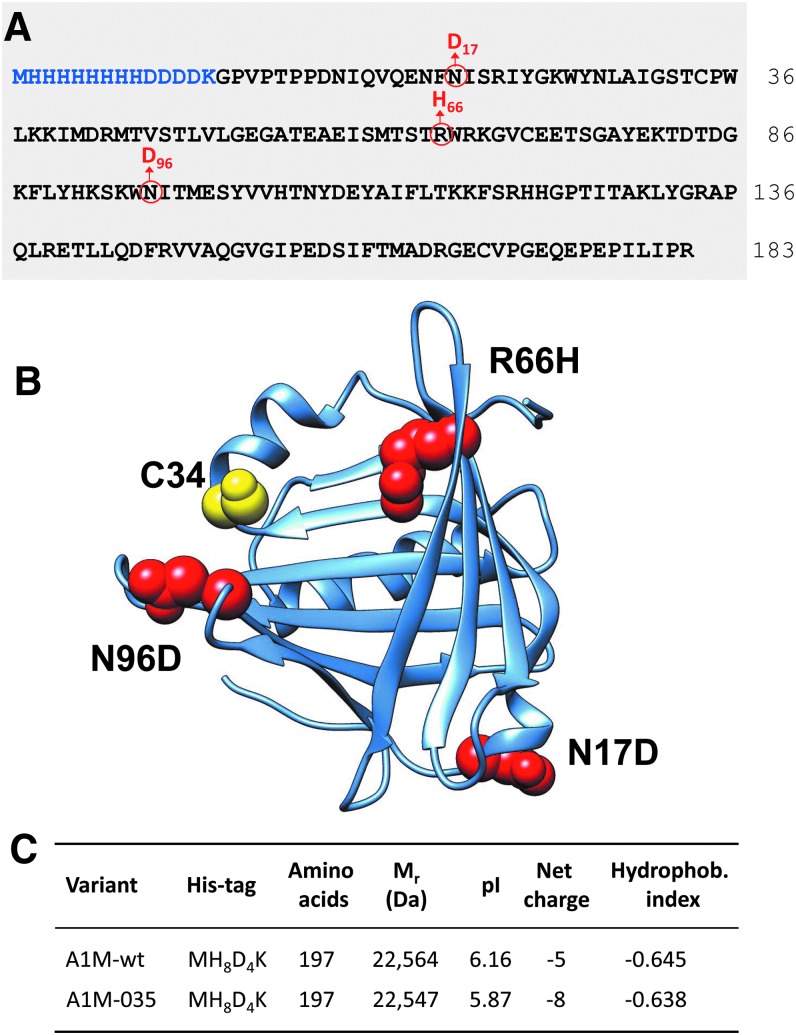
**Structure of rA1M-035.** **(A)** Amino acid sequence of rA1M. The N-terminal His-tag and linker are highlighted in *blue*. *Black letters* show the sequence of mature human plasma A1M (183 amino acids) and numbering is based on this sequence. The three amino acid substitutions of rA1M-035 are shown in *red*. The *blue* and *black letters* thus illustrate rA1M-wt, and the *blue*, *black*, and *red letters* show rA1M-035. Both variants encompass 197 aa. **(B)** Three-dimensional structure of rA1M based on the crystal data of trimmed rA1M, encompassing residues 8–163 of human A1M (28). The cysteine residue in position 34 (mutated to serine in the crystal structure), shown in *yellow*, is the critical site for the reductase, heme binding, and radical scavenging properties of A1M (3). The three substituted amino acids (N17, R66, N96) are shown in *red color* in their nonmutated forms. **(C)** Predicted M_r_, pI, net charge, and hydrophobicity index of the two rA1M variants. Since the net charge is pH-dependent, it was calculated at pH 7.4 where the H66 residue of rA1M-035 is expected to be >95% noncharged. A1M, α_1_-microglobulin; rA1M, recombinant human A1M, wt, wild-type. Data were obtained by the ProtParam Tool.

The design of rA1M-035 is shown in Figure 1A, B, illustrating the full amino acid sequence (Fig. 1A) and a model of the three-dimensional structure (Fig. 1B) built on the crystal structure of human A1M (28). The rationale of the design was initially to introduce a negative charge at the two N-glycosylation sites (N17, N96) in the absence of the two negatively charged N-glycans, which are present in human plasma A1M, but not in *E. coli* rA1M, and second to substitute R66 with a histidine, which is present in all rodent A1M-homologues. Theoretical values of molecular masses, pI, net charge at pH 7, and hydropathicity index of the tagged proteins are shown in Figure 1C. Due to the three amino acid substitutions, pI is lower, the net charge increased by three units, and hydropathicity decreased in rA1M-035 compared with rA1M-wt.

**FIG. 2.**
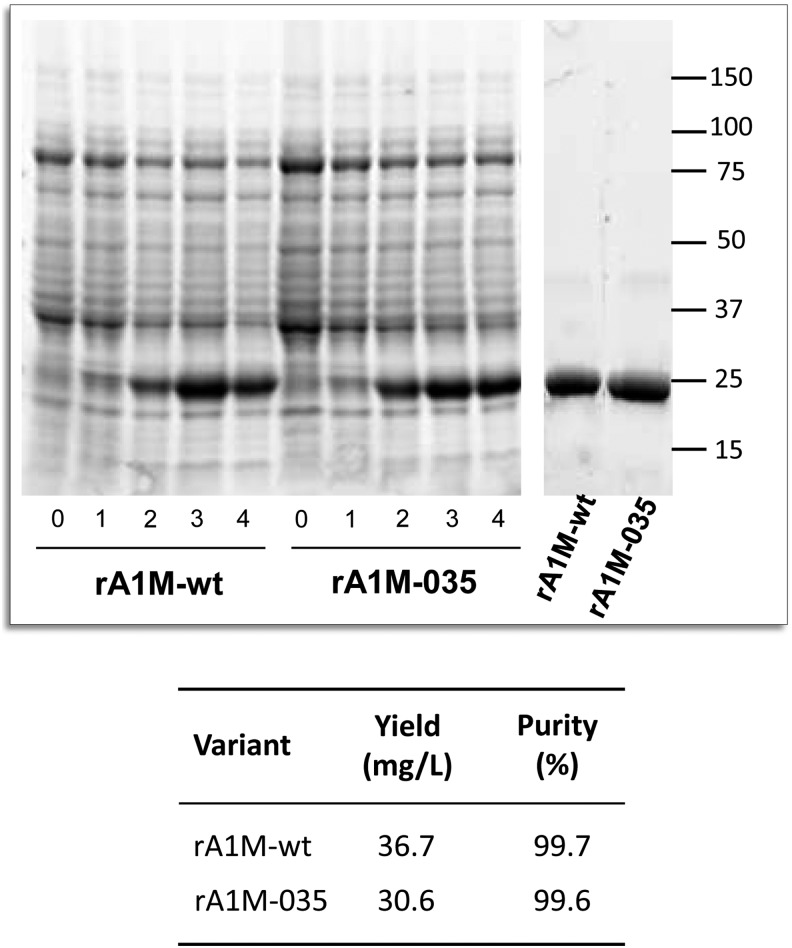
**SDS-PAGE of equal amounts of bacterial lysate from** ***Escherichia coli*** **cultures expressing rA1M-wt and rA1M-035, taken from uninduced cultures (0), and 1, 2, 3, and 4 h after induction with** **IPTG (*left panels*).** Ten micrograms of purified rA1M of both variants (*right panel*). The corresponding uncropped gels are shown in Supplementary Figure S1. The table shows the yields and purities of the purified proteins. Yield was calculated after protein determination by UV-absorbance at 280 nm, and purity was determined by densitometric analysis of SDS-PAGE bands. IPTG, isopropyl thiogalactoside; SDS-PAGE, sodium dodecyl sulfate/polyacrylamide gel electrophoresis.

**FIG. 3.**
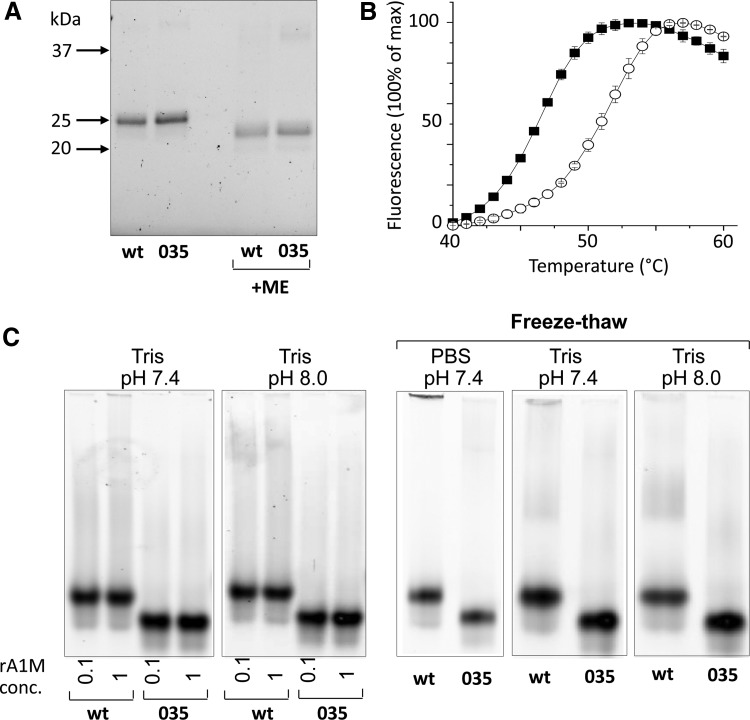
**Physicochemical properties of rA1M-variants. (A)** SDS-PAGE (12% gel) of 0.8 μg rA1M-wt and rA1M-035 under nonreducing and reducing conditions. The corresponding uncropped gel is shown in Supplementary Figure S2. **(B)** Thermostability of rA1M-wt (▪) and rA1M-035 (○) using differential scanning fluorimetry. Proteins were diluted to 0.1 mg/mL in 10 m*M* Hepes, 0.125 *M* NaCl, pH 8.0, containing 1:1000 SYPRO orange. Each point represents mean ± SD of three measurements. **(C)** Native PAGE of 20 μg rA1M-wt and rA1M-035. Two *left panels*: Proteins were transferred to 20 m*M* Tris-HCl +0.15 *M* NaCl, pH 7.4 or 8.0 and finally adjusted to 0.1 or 1 m*M*, and separated by native PAGE. Three *right panels*: The proteins were concentrated to 1 m*M* in PBS, pH 7.4, or 20 m*M* Tris-HCl +0.15 *M* NaCl, pH 7.4 or 8.0, subjected to one freeze/thaw cycle, and then separated by native PAGE. The corresponding uncropped gels are shown in Supplementary Figure S2. The percentage of large aggregates was calculated using densitometry analysis of the individual band intensities (Table 1). PBS, phosphate-buffered saline.

**FIG. 4.**
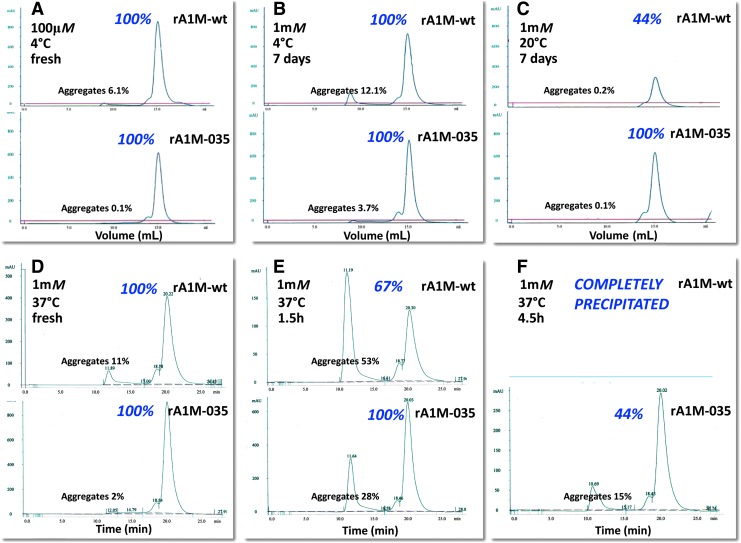
**Aggregation of rA1M-wt and rA1M-035 after storage at various temperatures and concentrations, analyzed by SEC.** Proteins were incubated at the indicated concentrations and time periods in PBS, centrifuged before application to a 24-mL Superose column, and eluted with 20 m*M* Tris-HCl, pH 8.0 + 0.15 *M* NaCl, at a flow rate of 0.1 mL/min. Monomeric, dimeric, and aggregated A1M eluted at **(A–C)** 15, 13.5, and 8 mL, respectively, or **(D–F)** 20, 18, and 12 min, respectively. Recovery of protein after storage, centrifugation, and SEC (shown in italics by the monomer peak) was calculated by comparing the total peak areas of treated *versus* nontreated samples. The percentage of large aggregates (shown above the aggregate peaks) was calculated from the area under the aggregate peak compared with the total peak area. SEC, size-exclusion chromatography.

Both variants were expressed with similar kinetics in 1 L cultures and purified to high purity with a yield around 30–40 mg/L culture (Fig. 2, corresponding uncropped gels shown in Supplementary Fig. S1; Supplementary Data are available online at www.liebertpub.com/ars). Protein size, cysteine arrangement, aggregation, and stability of the purified rA1M variants were analyzed by size exclusion chromatography (SEC), reversed-phase high-pressure liquid chromatography (HPLC), dynamic light scattering (DLS), differential scanning fluorimetry (DSF), reducing sodium dodecyl sulfate/polyacrylamide gel electrophoresis (SDS-PAGE), and native PAGE (Figs. 3 and 4; Table 1; uncropped native gels in Supplementary Fig. S2). Both variants showed the expected migration shift on SDS-PAGE under reducing/nonreducing conditions, suggesting one intrachain disulfide bridge (Fig. 3A, corresponding uncropped gel shown in Supplementary Fig. S2). Free thiol group analysis after reduction with DTT under native conditions resulted in 0.62 (SD = 0.09) and 0.67 (SD = 0.12) mol thiol groups per mol rA1M-wt and rA1M-035, respectively. This suggests that both proteins were folded correctly with formation of the C72-169 intrachain disulfide bridge and a free thiol group on C34. The two proteins displayed similar elution characteristics on HPLC and DLS radii, but rA1M-wt showed a higher tendency toward aggregation on PAGE and SEC, at 0.1 m*M* and pH 8.0 (Table 1,“unstressed”; Fig. 4). A higher T_m_ measured by DSF (Fig. 3B), and less aggregation measured by PAGE for rA1M-035, was seen at a high concentration (1 m*M*), lower pH (7.4), and freeze/thawing (Table 1,“stressed”; Fig. 3C). Furthermore, prolonged storage at 4, 22 ( = room temperature [RT]) and 37°C affected rA1M-035 less than rA1M-wt (Fig. 4). For example, rA1M-035 better tolerated storage at RT for a week, or at 37°C for 4.5 h, whereas 56% and 100% of rA1M-wt, respectively, were lost at these conditions.

**Table 1.** Physicochemical Properties of rA1M-wt and rA1M-035

*Conditions*^a^	*Concentrations (m*M*)*	*Methods*	*Parameters*	*rA1M-wt*	*rA1M-035*
Unstressed^b^	0.1	HPLC	Elution time (min)	12.1	12.1
			Monomer (%)	87	89
		DLS	Radius (nm)	2.9	3.2
		PAGE	Aggregates (%)	1.5	0.4
		SEC	Monomer (%)	87	93
Stressed					
Heat	0.1	DSF	T_m_ (°C)	46.6	51.6
High concentration	1	PAGE	Aggregates (%)	1.1	0.4
pH 7.4^c^	1	PAGE	Aggregates (%)	1.2	0.4
Freeze/thaw^b^	1	PAGE	Aggregates (%)	2.0	0.4
Freeze/thaw/PBS	1	PAGE	Aggregates (%)	13.2	7.6
Prolonged storage^d^					
4°C–1 day	1	SEC	Recovery^e^ (%)	100	100
			Aggregates (%)	17.9	3.9
4°C–7 days	1	SEC	Recovery (%)	100	100
			Aggregates (%)	12.1	3.7
room temperature–7 days	1	SEC	Recovery (%)	44	100
			Aggregates (%)	0.2	0.1
37°C–1.5 h	1	SEC	Recovery (%)	67	100
			Aggregates (%)	53	28
37°C–4.5 h	1	SEC	Recovery (%)	0	44
			Aggregates (%)	ND	15

^a^ Room temperature unless otherwise stated.

^b^ 20 m*M* Tris-HCl, 0.125 *M* NaCl, pH 8.0.

^c^ 20 m*M* Tris-HCl, 0.125 *M* NaCl, pH 7.4.

^d^ PBS.

^e^ Calculated after centrifugation, 10,000*g*, from total peak area compared to starting material.

A1M, α_1_-microglobulin; DLS, dynamic light scattering; DSF, differential scanning fluorimetry; HPLC, high-pressure liquid chromatography; ND, not determined; rA1M, recombinant human A1M; PAGE, polyacrylamide gel electrophoresis; PBS, phosphate-buffered saline; SEC, size exclusion chromatography.

Summarizing the physicochemical properties, rA1M-035 displayed a better tolerance to heat, high concentration, lower pH, and freeze/thawing than rA1M-wt.

### Binding, reduction, and antioxidation properties

Plasma, urine, and rA1M have previously been shown to have reductase activity and radical- and heme-binding properties, giving the protein an antioxidant protective function (3). Here, heme binding, reduction activity, and antioxidant capacity of rA1M-wt and rA1M-035 were compared. Overall, similar properties were obtained for the two rA1M-variants.

**FIG. 5.**
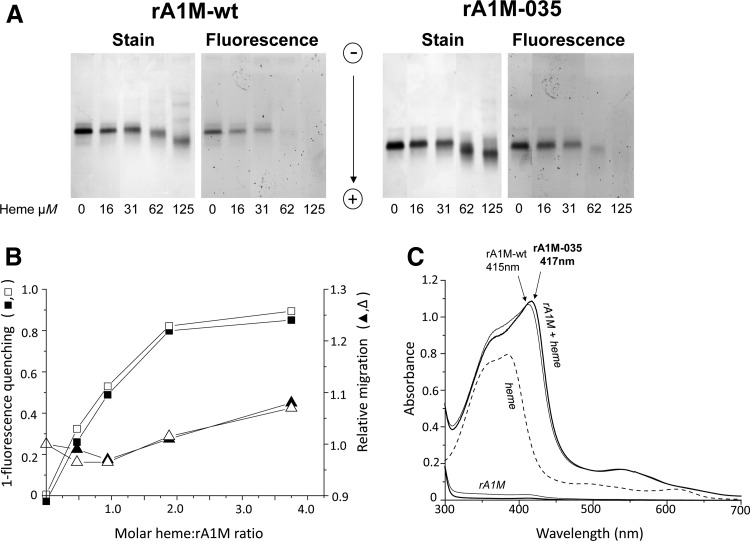
**Heme binding of A1M.** Heme binding analyzed by migration shift/fluorescence quenching on native **(A, B)** PAGE and **(C)** UV-absorbance spectrophotometry. **(A)** Fifteen micrograms of rA1M-wt or rA1M-035 were incubated with different amounts of heme for 30 min at 20°C, separated by native PAGE, and the gel analyzed by tryptophan fluorescence (fluorescence) and densitometry scanning after Coomassie staining (stain). The corresponding uncropped gel is shown in Supplementary Figure S3. **(B)** The images were digitalized by using Image Lab^™^ Software (Bio-Rad). Heme binding, measured as fluorescence quenching (*squares*) and migration distance (*triangles*), was plotted against the molar ratio A1M:heme. Mean values of duplicate experiments are shown, rA1M-wt (*filled symbols*), rA1M-035 (*open symbols*). **(C)** rA1M and heme were mixed (32 and 19 μ*M*, respectively), incubated for 2 h at 20°C, and scanned. The absorbance of the proteins (rA1M-wt, *solid line*; rA1M-035, *bold line*) and heme alone (32 and 19 μ*M*, respectively) are shown as comparison. The absorbance of the buffer (20 m*M* Tris-HCl, pH 8.0 + 0.15 *M* NaCl) was subtracted from all scans as blank.

The heme-binding was analyzed (Fig. 5) by using a combination of migration shift and fluorescence analysis (23). As a result of heme-incorporation, the migration of rA1M-wt and rA1M-035 in native PAGE analysis was slower at a heme:protein ratio <1, and faster at a heme:protein ratio >1, both variants showing the same dependence on heme-concentration (Fig. 5A, B; corresponding uncropped gels are shown in Supplementary Fig. S3). At high heme concentrations, the proteins also showed a tendency toward oligomerization, supporting previous findings for rA1M-wt (23). Likewise, heme-incorporation induced quenching of tryptophan fluorescence in both rA1M-wt and rA1M-035, with similar results (Fig. 5B). The coordination of heme in rA1M was previously shown to induce formation of a UV absorbance peak at 415 nm (23, 39). Similar UV absorbance patterns of rA1M-wt- and rA1M-035/heme complexes were seen (Fig. 5C). Heme binding was also analyzed by surface plasmon resonance (SPR) or BIAcore capturing rA1M on primary immobilized monoclonal anti-His tag antibodies. With a range of heme concentrations from 100 to 0.625 μ*M*, the SPR data were fitted to a 1:1 binding model as previously described for rA1M-wt (23), yielding kinetic rate constants k_a_ and k_d_ of 235 *M*^−1^s^−1^ and 3.72 × 10^−3^ s^−1^ for rA1M-wt, and 373 *M*^−1^s^−1^ and 1.66 × 10^−3^s^−1^ for rA1M-035, giving equilibrium binding constants K_D_ of 15.8 and 4.45 μ*M*, respectively. Thus, rA1M-035 displayed a slightly stronger binding and the K_D_ of rA1M-wt was in close agreement with the previously reported value of 13.8 μ*M* using the same method (23).

**FIG. 6.**
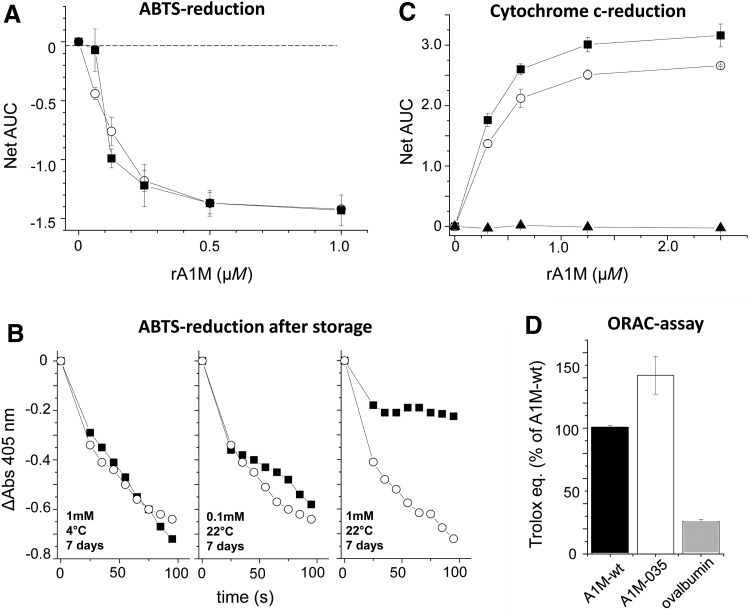
**Comparison of the reduction and antioxidation properties of rA1M-wt and rA1M-035. (A)** Freshly purified rA1M-wt (▪) or rA1M-035 (○) at various concentrations was mixed with ABTS-radical at 56 μ*M* in 25 m*M* sodium phosphate buffer pH 8.0 in microtiter plate wells, and the rate of reduction was followed by reading the absorbance at 405 nm during 95 s. The absorbance for each concentration was *plotted* against time and the AUC between 0 and 95 s was calculated for each concentration. The net AUC was calculated by subtracting the AUC of buffer only. Mean of triplicates ± SEM is shown. **(B)** The ABTS reduction rate was determined as described in (**A)**, but using rA1M-wt (▪) or rA1M-035 (○) after storage for 7 days at 4°C or at room temperature (RT) and the protein concentrations 0.1 or 1 m*M*. Single experiments are shown. **(C)** The reduction of cytochrome c was investigated by mixing dilution series (0–10 μ*M*) of rA1M-wt (▪) or rA1M-035 (○) with 100 μ*M* cytochrome c + 100 μ*M* NADH and following the increase in absorbance at 550 nm for 20 min. The assay was done in duplicate. The AUC was calculated for each concentration and the net AUC was calculated by subtraction of the AUC of buffer only. Data are presented as the net AUC ± SEM of two independent experiments. Ovalbumin was used as a negative control (▴). **(D)** The antioxidation ability was investigated in the ORAC assay. The activities of the rA1M-variants and ovalbumin at 5 μ*M* were compared to a Trolox standard and expressed as number of Trolox equivalents. Each assay was done in triplicate and the result of rA1M-wt was set to 100%. Data presented are the mean of two independent experiments ± SEM. ABTS, 2,2′-azino-bis-(3-ethylbenzothiazoline-6-sulfonic acid); AUC, area under the curve; ORAC, oxygen radical antioxidant capacity.

The rate of reduction of ABTS radicals (5) was similar for rA1M-wt and rA1M-035 using unstressed, freshly prepared proteins (Fig. 6A). After storage for 7 days at 1 m*M* and at 22°C, rA1M-wt displayed a slower reduction rate (Fig. 6B), suggesting a loss of protein activity in addition to the aggregation shown at these conditions (Table 1). The cytochrome c-reduction (8) was slightly slower for rA1M-035 (Fig. 6C), whereas the antioxidation capacity measured by the oxygen radical antioxidant capacity (ORAC) assay was somewhat higher for rA1M-035 (Fig. 6D).

### Cell protection capacity

**FIG. 7.**
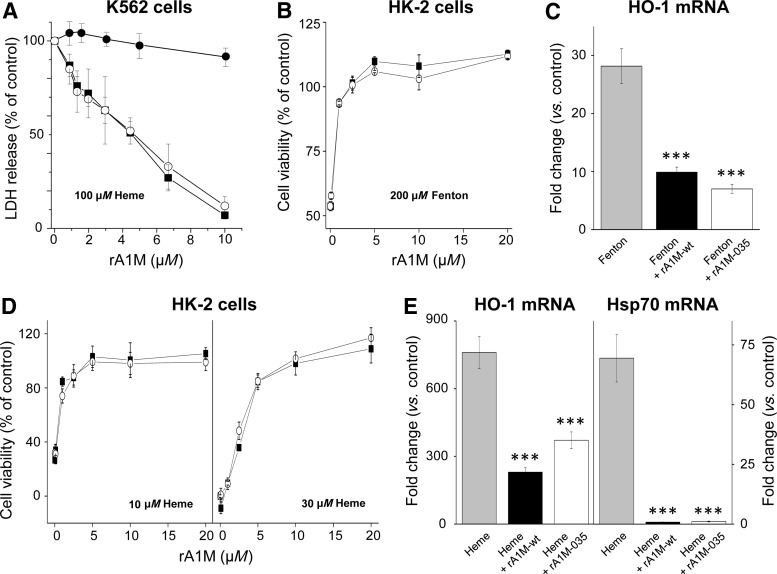
**Cell protection capabilities of rA1M. (A)** K562 cells, seeded at 10^5^ cells per well in a 96-well microtiter plate, were exposed to 100 μ*M* heme in the presence of a dilution series (0–10 μ*M*) of rA1M-wt- (▪), rA1M-035 (○), or ovalbumin (●) for 1 h. Cell death was monitored as release of LDH into the medium. The LDH value from live cells was subtracted and the signal of heme-incubated cells without rA1M was set to 100% and the values of the rA1M incubations were calculated in relation to this. The assay was made in duplicate. The average result from three independent experiments (mean ± SEM) is shown. **(B–E)** HK-2 cells were exposed to a mixture of 200 μ*M* (NH_4_)Fe(SO_4_)_2_, 400 μ*M* hydrogen peroxide, and 2 m*M* ascorbate (the Fenton reaction, **B** and **C**), 10 or 30 μ*M* heme (**D**), and 30 μ*M* heme **(E)** with or without the simultaneous addition of 0–20 μ*M* rA1M-wt (displayed as ▪ in **B** and **D**, and *black columns* in **C** and **E**) or rA1M-035 (displayed as ○ in **B** and **D**, and *white columns* in **C** and **E**) for 6 h. After incubation, cells were analyzed for cell viability using **(B** and **D)** WST-1 or **(C, E)** mRNA expression of HO-1 and Hsp70. The cell viability (**B** and **D**) was normalized against control samples from untreated cells. Results are from triplicate experiments and presented as mean ± SEM. The mRNA expression of HO-1 and Hsp70 **(C, E)** was normalized against GAPDH and is given as fold change. The fold-change values were calculated by normalizing against control samples from untreated cells. Results are from triplicate experiments and presented as mean ± SEM. Differences between the respective exposures and control conditions were analyzed using one-way ANOVA with *post hoc* Bonferroni correction. *Statistical comparison *versus* **(C)** Fenton or **(E)** heme. ***p < 0.001. No significant difference was observed when comparing rA1M-wt *versus* rA1M-035. GAPDH, glyceraldehyde-3-phosphate dehydrogenase; HO-1, heme oxygenase-1; LDH, lactate dehydrogenase.

It has previously been shown that rA1M-wt can inhibit heme-induced cell damage of the human erythroid cell line K562 (36). Here, the cell protection properties of rA1M-wt and rA1M-035 were tested in K562 cells and a human kidney proximal tubule epithelial cell line (HK-2), exposed to free heme and free iron. Figure 7A shows that both rA1M-variants completely inhibited cell death of K562 cells exposed to 100 μ*M* heme, measured as extracellular release of the cytosolic marker lactate dehydrogenase (LDH). The dose/response curves of rA1M-wt and rA1M-035 overlap almost completely, suggesting highly similar cell protection capacities. No inhibition was seen by the control protein, ovalbumin.

Protection of HK-2 cells is shown in Figure 7B–E. Cell damage was induced by the Fenton reaction, a mixture of free iron, ascorbate, and hydrogen peroxide, which generates hydroxyl radicals (Fig. 7B). Cell viability, measured by the WST-1 assay, was restored by both rA1M-variants, displaying overlapping dose/response curves. The upregulation of heme oxygenase-1 (HO-1), a well-documented biomarker of oxidative stress response (6), was significantly suppressed by rA1M-wt and rA1M-035 to similar degrees (Fig. 7C). Cell damage of HK-2 cells was also induced by exposure to heme and was shown to be inhibited by both rA1M-variants (Fig. 7D, E). Cell viability, measured by the WST-1 assay, using two heme concentrations, 10 and 30 μ*M*, was restored by both proteins to a similar degree (Fig. 7D). The upregulation of HO-1 and another cellular stress response gene, Hsp70, was inhibited to a similar degree by both rA1M-variants (Fig. 7E). Remarkably, the 75-fold upregulation of the latter gene was completely inhibited by both rA1M-variants.

### *In vivo* distribution in mice

**FIG. 8.**
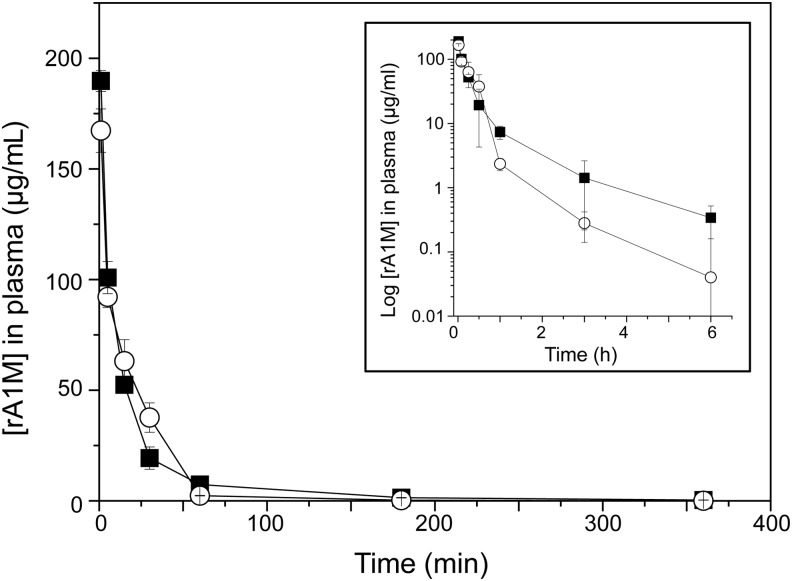
**Plasma clearance (pharmacokinetics) of rA1M-wt and rA1M-035 injected intravenously in animals.** Nonlabeled rA1M-wt (▪) or rA1M-035 (○) was injected in Wistar rats (5.1 mg/kg) and blood was collected at regular intervals. rA1M concentrations were determined by competitive RIA using the particular rA1M-variant as standard in each case. Each point represents three animals and is presented as mean ± SD. Inserted graph displays logarithmic scale on y-axis to highlight the pharmacokinetics in the late phase. RIA, radioimmunoassay.

Intravenously (i.v.) injected rA1M was previously shown to rapidly clear from blood and predominantly localize to the kidneys in rats and mice (1, 26). Here, we compared the amounts found in blood plasma and organs at different times after i.v. injection of rA1M-wt and rA1M-035 (Fig. 8; Table 2). Similar turnover rates were seen in plasma during the first 6 h postinjection (Fig. 8), with a fast clearance phase during the first 60 min (T_1/2_ = 2.1 min ±0.91 for rA1M-wt and T_1/2_ = 1.8 min ±0.33 for rA1M-035), and a slower clearance phase during the next 5 h for both rA1M-variants (Fig. 8B). Higher levels were seen for rA1M-wt than for rA1M-035, but the difference was not significant. The distribution of rA1M in the investigated organs after 10 and 30 min did not show any significant differences between the two proteins (Table 2). Both rA1M-wt and rA1M-035 were found predominantly in the kidneys, and smaller amounts were seen in the heart, liver, lung, skin, and spleen, while negligible amounts were found in the brain.

**Table 2.** Biodistribution of rA1M-wt and rA1M-035 After Intravenous Injection in Mice

*Organ*	*10 min μg/g organ (SD)*		*30 min μg/g organ (SD)*	
	*rA1M-wt*	*rA1M-035*	*rA1M-wt*	*rA1M-035*
Plasma	11. 9 (1.7)	7.7 (1.3)	1.19 (0.12)	1.10 (0.16)
Brain	0.185 (0.022)	0.137 (0.041)	0.028 (0.003)	0.032 (0.007)
Heart	3.39 (0.47)	2.39 (0.51)	0.79 (0.10)	0.47 (0.08)
Kidney	21.7 (4.1)	21.8 (4.5)	3.27 (0.22)	3.66 (0.32)
Liver	5.04 (0.77)	2.07 (0.27)	1.05 (0.09)	0.39 (0.10)
Lung	2.46 (0.27)	1.75 (0.30)	1.12 (0.11)	1.13 (0.23)
Skin	0.87 (0.07)	1.05 (0.07)	0.68 (0.15)	1.19 (0.17)
Spleen	3.09 (0.49)	1.37 (0.22)	0.50 (0.04)	0.24 (0.02)

wt, wild-type.

### *In vivo* protection of kidneys

**FIG. 9.**
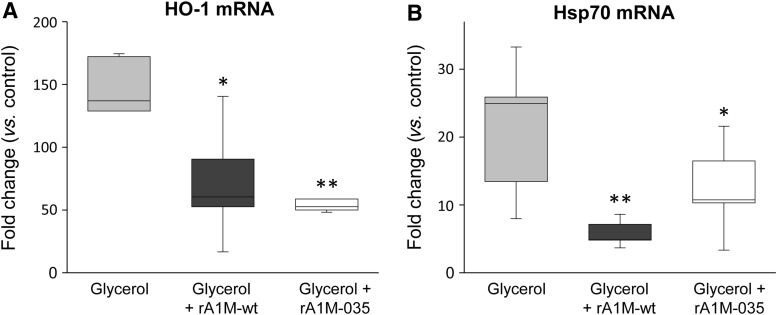
**Female C57BL/6 mice were exposed to glycerol (2.0 mL/kg, i.m.) followed by i.v. administration of rA1M-wt (*dark gray bars*,** ***n* = 10), rA1M-035 (*white bars*,** ***n* = 10), or vehicle buffer (sham control,** ***gray bars*,** ***n* = 6) 30 min postglycerol injections.** At 4 h (postglycerol administration), animals were euthanized and kidneys excised, snap-frozen, and subsequently analyzed for mRNA expression of **(A)** HO-1 and **(B)** Hsp70 using real-time polymerase chain reaction. mRNA expression was normalized against those of GAPDH, and fold change values were calculated by normalizing against control samples from untreated animals (controls). Results are presented as box plots, displaying medians and 25th and 75th percentiles. Statistical comparison between groups was performed by ANOVA with *post hoc* Bonferroni correction. *Statistical comparison *versus* glycerol. *p < 0.05, **p < 0.01. No significant difference was observed when comparing rA1M-wt *vs.* rA1M-035. i.m., intramuscular; i.v., intravenous.

Previously, rA1M has shown *in vivo* therapeutic effects in animal models where heme- and oxidative stress-related kidney injuries are induced (30, 42, 45). Here, we investigated the potential *in vivo* protective effects of the two rA1M-variants in a mild variant of the glycerol-induced rhabdomyolysis mouse model where AKI develops as a result of muscle rupture with release of myoglobin, heme, radicals, and other tissue components. A low glycerol dose was given, 2 mL/kg, and plasma creatinine/blood urea nitrogen (BUN)-levels did not display any significant changes (not shown). However, at 4 h postglycerol injection, rhabdomyolysis caused a massive renal increase of HO-1 and Hsp70 mRNA, two biomarkers of cellular stress (Fig. 9A, B). Administration of the rA1M-variants (7 mg/kg animal weight rA1M-wt or rA1M-035), 30 min after glycerol injection, significantly inhibited or reversed the upregulation of HO-1 and Hsp70 genes. No significant difference between the two rA1M-variants was seen at the applied dose.

## Discussion

In this work it was shown that a genetically modified variant of rA1M has improved physicochemical properties, allowing storage at higher concentrations, higher temperature, lower pH, and longer times compared with nonmodified rA1M (rA1M-wt). The new rA1M-variant, denoted rA1M-035, displayed the same binding and enzymatic capacities as rA1M-wt, showed therapeutic protective effects *in vitro* and potential for *in vivo* kidney protection. Therefore, rA1M-035 is the preferred candidate to be developed for clinical therapeutic use.

rA1M-035 is the end product of a screening project aimed to increase protein solubility and stability by introducing amino acid substitutions, while maintaining or improving the biological functions of A1M (Rosenlöf *et al.*, article under preparation). To achieve this, 36 rA1M-variants were expressed in *E. coli* together with rA1M-wt, purified, and analyzed for protein chemical and functional properties. First, rA1M from 11 species and 14 selected point mutations of human A1M were evaluated. None of the animal homologues, but a few of the rA1M-variants with point mutations, showed improved stability with maintained function. Second, the successful point mutations were combined into scrambled new combinations and with two different N-terminal extensions (“tags”), or without tags. Of these, the rA1M variant called “rA1M-035,” with an N-terminal MH_8_D_4_K-tag, displayed the best solubility/stability properties. Finally, this variant was tested side-by-side with rA1M-wt, equipped with the same N-terminal tag, in physicochemical and functional assays, the result of which is presented in this article. During the initial screening phase, a few versions of the N-terminal His-tag were compared to nontagged rA1M. A clear advantage of an N-terminal His-tag on solubility, stability, and expression levels of both rA1M-variants was seen. The structural peptide extension also allows specific detection and quantification of infused/injected rA1M in humans without interference of endogenous A1M. Although it may be argued that the N-terminal His-tag may be immunogenic *in vivo*, its contribution to physicochemical protein stability strongly favors a future A1M-based drug that includes the His-tag.

Mostly, the analysis of rA1M-035 properties was done using methods and experimental setups that previously have been applied to evaluate recombinant rA1M-wt, plasma A1M, or urine A1M, and where the results have been published (1, 5, 8, 23, 24, 36, 39). The purpose of this study was to compare the performance of the new rA1M-variant to the expected outcome of rA1M-wt. However, to study some of the functional properties of rA1M-035, a novel approach and development of new methods were needed. In these cases, the performance of rA1M-wt was not known and the results are therefore novel, that is, not previously published for either rA1M-wt or rA1M-035. Such novel results are discussed below and include the ORAC assay (Fig. 6D), the WST assay of cell proliferation (Fig. 7B, D), protection of HK-2 cells (Fig. 7B–E), blood clearance kinetics (Fig. 8), and the suppression of kidney stress gene response in the *in vivo* mouse rhabdomyolysis model (Fig. 9).

The rA1M-035 variant displayed improved protein stability compared with rA1M-wt. The lower stability of rA1M-wt is seen as a tendency to aggregate into large complexes in buffers with pH <8.0, temperatures above 10°C, concentrations above 0.1 m*M*, prolonged storage, and freeze/thawing. For example, heating of a 1 m*M* solution of rA1M-wt in phosphate-buffered saline (PBS) to 37°C resulted in complete precipitation within 4.5 h (Fig. 4; Table 1). As described in this work, rA1M-035 showed better performance, that is, less aggregation and precipitation at these conditions. Heating of a 1 m*M* solution of rA1M-035 in PBS to 37°C resulted in 44% recovery with only small amounts of aggregates. The relatively low stability of rA1M-wt may be explained, in part, by the lack of glycosylation with the negatively charged, highly hydrophilic N-glycans of human A1M at amino acid positions N17 and N96 (9, 17). The glycans probably prevent intermolecular hydrophobic interactions, which may lead to aggregation. When expressing the human A1M gene in *E. coli*, the noncharged side-chains of N17 and N96 are exposed on the surface of the protein instead of the strongly hydrophilic glycans, leading to lower stability and aggregation of rA1M-wt. Replacing N17 and N96 with negatively charged D17 and D96 therefore results in a lower degree of hydrophobic intermolecular interactions and better stability. The R66H mutation of rA1M-035 also resulted in improved stability compared with an rA1M-variant with only the N17D and N96D substitutions (Rosenlöf *et al.*, article under preparation). The structural explanation for the improved stability of properties inferred by this substitution is not known, but the rationale for introducing this substitution is that it is seen in all rodent species.

An ideal A1M-based drug for therapeutic use should be stable on storage at <40°C for long time periods, preferably freeze-dried, have no aggregation tendencies, and be easily dissolved at millimolar concentrations in water. This is actually an opposite description of the physicochemical properties of human plasma A1M. However, purification of A1M from human plasma for drug use is not feasible today, since low yield and scarcity of raw material would make production costs unrealistic. It is of course possible to further optimize rA1M, beyond that of rA1M-035, to achieve even higher stability and solubility, improved functional antioxidation protection properties, or even tailor-made rA1M-variants as therapeutic drugs for certain disease groups. For example, an rA1M-species with higher affinity for heme-groups may be more efficient for treatment of sickle-cell disease. rA1M-035 was developed by targeted mutagenesis, and possible future strategies include screening of libraries from random mutagenesis and/or short A1M peptides.

Overall, the two rA1M-variants displayed similar heme-binding and enzyme activities. Minor differences were seen, although not in a consistent manner. Whereas the reduction of cytochrome c was approximately 15% slower for rA1M-035 (Fig. 6C), the antioxidant capacity, as measured by the ORAC assay, of rA1M-035 was increased by approximately 40% (Fig. 6D). It is possible that the amino acid substitution Arg→His in position 66, located inside the lipocalin binding pocket (Fig. 1), may influence the performance of rA1M in these assays. It was shown previously that three Lys residues in positions 92, 118, and 130, which are located inside the upper part of the pocket similar to position 66, have impact on the heme-binding (39) and cytochrome c-reduction rate (8). However, rA1M-035 displayed the same, perhaps somewhat better, heme-binding capacity and antioxidation capacity. Overall, the two rA1M-variants were found to be similar with regard to heme-binding and functional activities.

The pharmacokinetics analysis suggests a rapid clearance rate of i.v.-injected rA1M from blood, with a half-life in blood around 2 min during the first 60 min and a slower clearance after 1 h. After 6 h, less than 1% of both rA1M-variants remained in the blood circulation. The rapid turnover of the injected rA1M supports previous data on plasma A1M and rA1M expressed in insect cells (26, 44). As discussed previously (1, 3), the fast clearance of A1M reflects a distribution of the protein to its physiological targets, that is, extravascular space, extracellular matrix, and damaged epithelial cells. In other words, the short half-life of injected A1M is a prerequisite for an optimal drug delivery. Most of the injected rA1M was localized to the kidneys, followed by the liver, and no significant difference could be observed between rA1M-035 and rA1M-wt. The biodistribution agrees with previous results obtained with rat urinary A1M and human rA1M, injected in rats and mice, respectively (1, 26).

It has previously been shown that rA1M-wt can inhibit heme-induced cell death of the human erythroid cell line K562 (36). Almost complete inhibition (∼70%) was demonstrated at a molar A1M:heme ratio of 1:20. Here, this was confirmed, that is, both rA1M-wt and rA1M-035 could almost completely inhibit heme-induced cell death at a molar A1M:heme ratio of 1:10 for K562 cells, and 1:6 for HK-2 cells (Fig. 7A, B). This suggests that the protection mechanisms are not exclusively dependent on heme binding, but may involve several of the mechanisms demonstrated for A1M, *c.f.* scavenging of downstream ROS generated by the free heme group, reduction of oxidation lesions, as well as direct binding of heme.

The results show *in vitro* and *in vivo* therapeutic effects of rA1M on kidney cells. Both rA1M-variants protected kidney tubular epithelial cells (HK-2) against oxidative stress-induced insults. The results support and extend previous data showing protection by rA1M-wt of blood cells, liver cells, and skin cells cultured under oxidative stress (31, 35, 36). Figure 7 shows a complete rescue of HK-2 cells from cell death with both rA1M-wt and rA1M-035 after exposure to hydroxyl radicals generated by Fenton reaction or exposure to free heme. Similar potencies of the two variants were obtained. The oxidative stress induced to the cells was also measured as an upregulation of the two stress response genes HO-1 and Hsp70, with their main functions in heme-degradation/antioxidation and unfolded protein response, respectively (6, 13, 16). The upregulation of both genes was significantly inhibited by the addition of either of the rA1M-variants, suggesting clear cell protective effects of both. Furthermore, i.v. injection of rA1M *in vivo* resulted in protection of kidneys, for example, reduced mRNA expression of the stress response genes HO-1 and Hsp70, using a mild variant of mouse rhabdomyolysis-induced AKI (Fig. 9). In this model, myolysis of the thigh muscle induces release of cell debris, including mitochondria, nuclear content, and membrane fractions, free myoglobin, and myoglobin-derived heme. These factors generate free radicals and induce oxidative stress and tissue and vascular damage (29, 43). The observed AKI is therefore believed to largely be a result of oxidative stress and as shown in Figure 9, the two markers of oxidative stress, HO-1 and Hsp70, are massively upregulated in the kidney tissues 4 h after glycerol injection. The upregulation was reversed by both rA1M-variants, suggesting that the protein has potential antioxidation effects on kidneys *in vivo*. These effects can probably be explained both by the heme and radical binding as well as reductase activities of A1M, plus the fact that a large proportion of the i.v.-injected rA1M is localized to the kidneys. Of note, in our mild model of rhabdomyolysis-induced kidney damage, using 2 mL/kg glycerol, we did not observe an increase in serum creatinine or BUN, and therefore, the model might not reflect all aspects of AKI.

## Materials and Methods

### Expression and purification of rA1M proteins

The genes coding for the two rA1M variants were synthesized and cloned into their PJ401express vector (T5 promoter, kanamycin resistance) by DNA2.0, Inc., USA (new name: ATUM). The rA1M-wt gene codes for an N-terminal MHHHHHHHHDDDDK-extension followed by the human 183-amino acid A1M-sequence (24). The rA1M-035 gene (Fig. 1A, B) codes for the same N-terminal His-tag extension followed by the human A1M sequence with three amino acid substitutions (N17D, R66H, and N96D). The DNA sequences were confirmed by sequencing. The vectors were transformed into BL21 Star(DE3) (Invitrogen, Life Technologies, Carlsbad, CA).

The rA1M expression clones were grown in complete NYAT [15 m*M* (NH_4_)_2_SO_4_, 84 m*M* K_2_HPO_4_, 23 m*M* NaH_2_PO_4_, 2.2 m*M* (NH_4_)_2_-citrate, 1% (w/v) glucose, 2 m*M* MgSO_4_, 9 μ*M* CaCl_2_, 85 μ*M* FeCl_3_, 1.3 μ*M* ZnSO_4_, 1.3 μ*M* CuSO_4_, 1.8 μ*M* MnSO_4_, 1.5 μ*M* CoCl_2_, 108 μ*M* EDTA, 50 μg/mL kanamycin] to an OD_600_ of 1.5. Protein expression was then induced by addition of 1 m*M* isopropyl thiogalactoside (IPTG). The production continued for 4 h. Samples for SDS-PAGE analysis were taken before induction, and 1, 2, 3, and 4 h after induction (Fig. 2).

rA1M-wt and rA1M-035 were purified from *E. coli* culture inclusion bodies and refolded essentially as described (24). The refolded protein was then separated by ion-exchange chromatography on an ÄKTA purifier 10 instrument (GE Healthcare) using a 5 mL Bio-Scale Mini UNOsphere Q Cartridge (Bio-Rad Laboratories, Hercules, CA), equilibrated with 20 m*M* Tris-HCl pH 8.0, washing with five column volumes of 20 m*M* Tris-HCl pH 8.0 before elution with a 20-column volume linear gradient from 0 to 0.35 *M* NaCl. The rA1M-containing fractions were pooled and concentrated to 100 μ*M*. Endotoxins were removed by passage through EndoTrap^®^ blue resin (Hyglos GmBH, Bernried, Germany) according to the manufacturer's description. The endotoxin content, determined using the Limulus Amebocyte Lysate test kit (LAL QCL-1000, Lonza) according to the manufacturer's description, was always below 1EU/mg protein. Final protein solutions were sterile filtered and frozen at −20°C in aliquots and stored at −80°C.

### Gel electrophoretic analysis of size and purity

SDS-PAGE was run using standard protocols. Proteins were separated on stain-free 4–20% Criterion TGX^™^ gels (Bio-Rad) at 300 V for 17 min, with or without 2% mercaptoethanol in the sample buffer. Native PAGE was run without SDS and without reducing agents on stain-free Criterion 4–20% TGX gels at 200 V for 40 min. The gel bands were analyzed on a ChemiDoc MP instrument (Bio-Rad) and quantified using Image Lab^™^ Software (Bio-Rad).

### Thiol determination

The molar contents of free thiol groups in A1M were determined using the thiol and sulfide quantitation assay kit (cat. No. T6060; Thermo Fisher Scientific, Waltham, MA) according to instructions from the manufacturer. Before analysis, 100 μg of each protein was reduced by incubation with 10 m*M* DTT in 0.1 *M* Tris-HCl pH 8.0 for 1 h, and dialyzed exhaustively.

### Analytical chromatography

Proteins were analyzed by size exclusion on an ÄKTA purifier 10 instrument using a 24-mL Superose 12 10/30 GL column (GE Healthcare, Little Chalfont, United Kingdom). The column was equilibrated with 20 m*M* Tris-HCl, pH 8.0 + 0.15 *M* NaCl, using a flow rate of 1 mL/min. Protein, 100–200 μg, was loaded onto the column in a volume of 100 μL and eluted with 20 m*M* Tris-HCl, pH 8.0 + 0.15 *M* NaCl, using a flow rate of 0.75 mL/min. Typically, monomeric rA1M was eluted after 15 mL/20 min, dimeric after 13–14 mL/18–19 min, and large aggregates were eluted after 8 mL/12 min. The percentage of large aggregates was calculated from the area under the 8-mL peak compared with the total peak area. The percentage of total protein retrieved on the column after stress treatment (Fig. 4; Table 1) was calculated by comparing the total peak areas of treated *vs*. nontreated samples.

Reversed-phase HPLC was run on an Agilent 1260 Infinity Binary LC system using an Aeris Widepore 3.6 μ*M* XP-C8 column (Phenomenex, Inc.). The column was run at 25°C using a flow rate of 1 mL/min and equilibrated with a mixture of 70% H_2_O + 0.1% trifluoroacetic acid (TFA) and 30% acetonitrile +0.1% TFA. Ten microliters ( = 10 μg of protein) were loaded and eluted with a linear gradient of 30–50% acetonitrile over 20 min. Washing with 95% acetonitrile for 10 min regenerated the column.

### Dynamic light scattering

DLS analysis of nonstressed and shearing-stressed samples was done using the service of SARomics Biostructures AB, Lund. rA1M samples, diluted to 10 μ*M* in 10 m*M* Tris-HCl, pH 8.0 + 0.125 *M* NaCl, were analyzed on a Malvern APS instrument (Malvern Instruments Ltd., Malvern, United Kingdom) at 20°C. Samples were prepared in duplicate and each sample was monitored three times.

### Differential scanning fluorimetry

Thermostability of the rA1M-variants was analyzed by DSF using the service of SARomics Biostructures AB, Lund. A1M diluted to 4.4 μ*M* in 10 m*M* HEPES, pH 8.0 + 0.125 *M* NaCl, was mixed with SYPRO orange (1000 × dilution of SYPRO orange in total). The analysis was made in duplicate and the average melting temperature (T_m_) was calculated.

### Mechanical stress induction

Ten microliters of 100-μ*M* rA1M solution was exposed to shearing force stress by 80 pipettings with a multiple channel pipette using 0–10 μL pipette tips. The stress treatment was performed in duplicate. After pipetting, the duplicates were combined and diluted 10 times before analysis with DLS as described.

### High-concentration stress induction

Solubility and stability of rA1M were analyzed at a high concentration. Five hundred microliters of 100 μ*M* rA1M solution was concentrated 10-fold to 50 μL using Amicon Ultra-0.5, 10K devices (Merck Millipore, Billerica, MA) by centrifugation at 14,000*g* for 10 min. After concentration, the volumes were corrected to exactly 50 μL using the respective flow through. Concentrated and nonconcentrated samples (10 μg) were compared side-by-side on native PAGE. The influence of different buffers was examined by diafiltration of the samples before concentration. This was done by five cycles of 10-time dilution/concentration in Amicon Ultra-15, 10K devices.

### Heme binding

Heme binding was analyzed as previously described by native PAGE (23), UV-spectrophotometry (39), and SPR (23). Briefly, for native PAGE, rA1M and various concentrations of heme were incubated in Tris-buffer, pH 8.0, for 30 min at RT, separated by native PAGE on stain-free 12% Criterion TGX gels at 200 V for 40 min. The gel bands were analyzed on a ChemiDoc MP instrument (Bio-Rad) for tryptophan fluorescence using the “stain-free” setting, stained with Coomassie brilliant blue, destained, and imaged again on the ChemiDoc using the Coomassie setting. Both sets of bands were then quantified using Image Lab Software (Bio-Rad). Heme binding was then estimated as the amount of quenching of the tryptophan fluorescence relative to total protein amounts after Coomassie staining. Absorbance spectra were measured on a Beckman DU 800 spectrophotometer (Beckman Instruments, Fullerton, CA) using a scan rate of 600 nm/min in the UV-Vis region between 250 and 700 nm at 22°C. Concentrations of rA1M and heme were 32 and 19 μ*M*, respectively, in 20 m*M* Tris-HCl, pH 8.0, 0.15 *M* NaCl. Heme was added from a stock solution of 10 m*M* in dimethyl sulfoxide (DMSO). Protein solutions were scanned 2 h after mixing. SPR experiments were performed using BIAcore X100 (GE Healthcare, Uppsala, Sweden). Approximately, 10,000 response units of anti-His mouse IgG1 monoclonal antibody (cat. No. MAB 050; R&D Systems, Minneapolis, MN) were immobilized to CM5 chips by amine coupling, rA1M was captured, heme samples (100–0.625 μ*M* in 50 m*M* Na-phosphate, pH 8.0 + 1% DMSO) injected, data fitted, and calculations performed as described (23).

### ABTS reduction assay

The assay is modified from (5) and measures the rate of reduction of the synthetic radical ABTS. ABTS di-ammonium salt (Sigma-Aldrich), 7 m*M*, was oxidized with 2.45 m*M* K_2_S_2_O_8_ overnight and then diluted to 56 μ*M* in 25 m*M* sodium phosphate buffer pH 8.0. One hundred microliters of the ABTS working solution was added per well in a 96-well plate. A time point zero measurement was done at 405 nm using a PerkinElmer Plate reader, model 550 (PerkinElmer, Massachusetts). Two microliters of an rA1M solution (0–100 μ*M*) was quickly added using a multichannel pipette. The kinetics of the decrease in absorbance at 405 nm was quickly followed for 95 s. For practical reasons, only eight wells were analyzed at the time. The rA1M dilution series was run in duplicate or triplicate. If the number of samples to be analyzed required several plates, new ABTS stock was diluted into working stock for each plate and an rA1M-wt reference sample was included in all plates. The absorbance for each concentration was plotted against time and the area under the curve (AUC) was calculated for each concentration. The net AUC was calculated by subtracting the AUC of buffer only.

### ORAC assay

The commercial kit OxiSelect™ ORAC activity assay (Cell Biolabs, Inc.) is based on the oxidation and destruction of a fluorescent probe by peroxyl radicals. When an antioxidant is present, this destruction is inhibited. As standard, the water-soluble vitamin E derivate, Trolox, is used. Performance, analysis, and calculations followed the kit manual and an rA1M concentration of 2.5–5 μ*M* fitted nicely into the standard curve. Ovalbumin (Sigma-Aldrich, St. Louis, MO) was used as a negative protein control.

### Cytochrome c reduction assay

The assay, which measures the kinetics of the reduction of the heme chelated iron atom in cytochrome c, is modified from Allhorn *et al.* (8). A working solution was made by mixing 100 μ*M* cytochrome c (Sigma-Aldrich) and 100 μ*M* NADH in 10 m*M* Tris-HCl, pH 8.0 + 0.125 *M* NaCl. Eleven microliters of an rA1M solution (0–100 μ*M*) was added to a 96-well plate in duplicate. Thereafter, 100 μL of the cytochrome c working solution was quickly added to each well using a multichannel pipette. The kinetics of the increase in absorbance at 550 nm was followed for 20 min. One plate was analyzed at the time. No biases over time could be observed. If several plates were to be analyzed, the same working solution was used for all without any observable artifacts caused by this procedure. After measurements, the results were analyzed as described for the ABTS assay.

### Protection of K562 cells

rA1M was previously shown to inhibit heme-induced cell death of human erythroid K562 cells (36). The cells were cultured in Dulbecco's modified Eagle's medium (DMEM) with GlutaMAX +10% fetal calf serum (FCS) and antibiotics (Gibco, Life Technologies Corp.) according to the instructions at ATCC^®^. Cells were washed and resuspended in DMEM without phenol red and FCS but supplemented with GlutaMAX I and antibiotics (Gibco). Cells were seeded into 96-well plates, 10^5^ cells per well, and exposed to 100 μ*M* heme in the presence of a 0–10 μ*M* rA1M or ovalbumin dilution series. A 10 m*M* stock solution of heme was prepared immediately before the experiment by dissolving ferriprotoporphyrin IX chloride (Porphyrin Products, Inc., Logan, UT) in DMSO (Sigma-Aldrich). As a positive control for cell death, 10 μL of lysis solution from the LDH detection kit was added. The cells were incubated in a 37°C CO_2_ incubator for 1 h. The plates were quickly centrifuged at 350*g* for 4 min before 50 μL of the medium was transferred to a 96-well microplate for analysis of LDH release using the CytoTox 96^®^ Non-Radio Cytotoxicity Assay (Promega Biotech AB, Nacka, Sweden) according to the manufacturer's instructions. Heme-induced cells typically gave two to three times higher signals compared with live cells and 70% of the signals of completely lysed cells. The average signal of cells incubated without heme, rA1M, or ovalbumin addition was subtracted from all and the signal of heme-only incubated cells was set to 100%. All other signals were related to this value. This procedure enabled comparison of several independent experiments.

### Protection of HK-2 cells

Human kidney cortex proximal tubule epithelial cells (HK-2, ATCC CRL-2190, ATCC, United Kingdom) were cultured in keratinocyte serum-free medium supplemented with bovine pituitary extract (0.05 mg/mL) and epidermal growth factor (5 ng/mL) (all from Invitrogen, United Kingdom). When cells reached ∼80–90% confluence, heme (0–30 μ*M*, from a freshly prepared 10 m*M* stock solution) or a mixture of (NH_4_)Fe(SO_4_)_2_, hydrogen peroxide, and ascorbate (0–200 μ*M*, the Fenton reaction), with or without the simultaneous addition of rA1M (0–20 μ*M*, wt- or rA1M-035), was added and cells were incubated for 6 h. After incubation, cells were analyzed for cell viability using WST-1 (the measured metabolic activity of cells, *e.g.*, measurement of cellular cleavage of the WST-1 stable tetrazolium salt to the soluble formazan dye, is a direct correlate to the number of viable cells) (Roche Diagnostics GmbH, Mannheim, Germany) according to the instructions from the manufacturer. The cell viability was normalized against control samples from untreated cells. Parallel cultures were harvested using QIAzol™ lysis reagent for RNA extraction (QIAGEN, Germantown, MD). Total RNA was extracted from cells to evaluate mRNA expression as described below.

### Plasma clearance and biodistribution

These studies were approved by the ethics committee for animal studies in Malmö-Lund, no. M21–15. For plasma clearance studies, each nonlabeled rA1M-variant was injected i.v in six male Wistar rats (5.1 mg/kg; stock solutions in 20 m*M* Tris-HCl, pH 8.0) and blood samples were taken in EDTA tubes at 1, 5, 15, 30 min, and 1, 3, 6 h postinjection, using different sampling intervals in groups of three rats to avoid oversampling. Plasma was aspirated after centrifugation 140*g* for 10 min, and the concentration of rA1M was determined by radioimmunoassay (RIA) as described (19), using the specific rA1M-variant as standard. The RIA is competitive, that is, measures the degree of displacement of ^125^I-labeled human A1M by the nonlabeled A1M in the unknown samples. The RIA reacted with human urinary A1M, rA1M-wt, and rA1M-035 equally well. For the biodistribution studies, the rA1M-variants were injected i.v. in C57BL/6NRj mice (5.0 mg/kg; stock solutions in 20 m*M* Tris-HCl, pH 8.0). The mice were sacrificed 10 min postinjection (*n* = 3) and after 30 min (*n* = 3). Organs were sampled, weighed, and homogenized in 5:1 (vol:weight) cell extraction buffer (cat. No. FNN0011; Invitrogen), containing 50 μL/mL complete Mini-EDTA-free proteinase inhibitor cocktail tablets (cat. no. 11836170001; Roche). A1M concentrations were determined by an in-house sandwich ELISA. Briefly, 96-well microtiter plates were coated overnight at 4°C with mouse monoclonal anti-A1M (clone 35.14, 5 μg/mL in PBS), washed, and then incubated with A1M standards [human urinary A1M, purified as described at our laboratory (11)] or homogenized tissue samples, diluted in incubation buffer (PBS +0.05% Tween 20 + 0.5% bovine serum albumin) for 60 min at RT. After washing, the wells were incubated with horseradish peroxidase-conjugated mouse monoclonal anti-A1M (clone 57.10, 5 ng/mL in incubation buffer) for 60 min at RT. The plates were washed and developed by incubating with SureBlue TMB Microwell Peroxidase Substrate (KPL) in the dark for 20 min and finally stopped with 1 *M* sulfuric acid. Absorbance was read at 450 nm in a Wallac 1420 Multilabel Counter. The two mouse monoclonal antibodies were raised against human urinary A1M by Agrisera AB, Sweden. The ELISA was specific for human A1M, did not cross react with endogenous mouse plasma A1M at the relevant concentrations, and reacted with human urinary A1M, rA1M-wt, and rA1M-035 equally well.

### Rhabdomyolysis-induced kidney damage

This study was approved by the ethics committee for animal studies in Malmö-Lund, no. M21–15. Female C57BL/6 mice with a body weight of 20.5 ± 0.7 g were obtained from Taconic (Ejby, Denmark), housed in type III cages with wire lids, at a constant room temperature with 12-h light/dark cycles. Temperature (20°C ± 0.5°C) and relative moisture (50 ± 5%) were maintained throughout the studies. All animals had free access to food [RM1(E) SQC; SDS, Essex, United Kingdom], tap water, and cage enrichment. After overnight water deprivation (15 h), animals were weighed, anesthetized using isoflurane, and then allocated to the following four groups: (i) control (*n* = 6), received no intramuscular (i.m.) or intravenous (i.v.) administration; (ii) glycerol (*n* = 10), received 50% sterile glycerol (Teknova, Hollister, CA) i.m. (2.0 mL/kg body weight, single dose, divided into both hind limbs); (iii) glycerol + rA1M-wt (*n* = 10), received rA1M-wt i.v. (7 mg/kg body weight, single dose) 30 min after glycerol i.m. (2.0 mL/kg body weight) administration; and (iv) glycerol + rA1M-035 (*n* = 10), received rA1M-035 i.v. (7 mg/kg body weight, single dose) 30 min after glycerol i.m. (2.0 mL/kg body weight) administration. Following i.m. administration, animals were placed on a heat pad during awakening and then put back to their cages and supplied with free access to food and water. After 4 h (postglycerol injection), animals were anesthetized using isoflurane and kidneys collected for RNA extraction followed by mRNA evaluation as described below.

### RNA isolation and real-time polymerase chain reaction

Total RNA was isolated from HK-2 cells, using Direct-zol™ RNA MiniPrep supplied by Zymo Research (Irvine, CA), or mouse kidneys, using NucleoSpin RNA/Protein (Macherey-Nagel, Duren, Germany) followed by RNeasy^®^ Mini Kit (QIAGEN, Germantown, MD). The OD ratio (optical density at 260 nm/280 nm) of RNA was always higher than 1.9. Reverse transcription was performed according to the manufacturer on 1.0 μg total RNA using iScript™ cDNA Synthesis Kit (Bio-Rad). RT^2^ qPCR Primer Assay [human (HK-2 cells) and mouse (kidneys) primers from QIAGEN] were used to quantify the mRNA expression of heme oxygenase 1 (HO-1) and heat shock protein 70 (Hsp70). Data were normalized to glyceraldehyde-3-phosphate dehydrogenase (human and mouse kidney, respectively, RT^2^ qPCR Primer Assay from QIAGEN). Data are presented as columns, displaying mean ± SEM, for *in vitro* data and box plots, displaying medians and 25th and 75th percentiles, for *in vivo* data. The fold change values were calculated by normalizing against control samples from untreated cells or animals (controls). Expression was analyzed using iTaq™ Universal SYBR^®^ Green Supermix (Bio-Rad). Amplification was performed as described by the manufacturer (Bio-Rad) for 40 cycles in an iCycler Thermal Cycler (Bio-Rad) and data analyzed using iCycler iQ Optical System Software (Bio-Rad).

### Statistics

Comparisons between unrelated groups were performed by ANOVA with *post hoc* Bonferroni correction. *p*-Values <0.05 were considered significant. T_1/2_-values for blood clearance determination were obtained by linear regression analysis of the slopes of log-lin diagrams.

## Supplementary Material

Supplemental data

Supplemental data

Supplemental data

## Abbreviations Used

A1M: α_1_-microglobulin
ABTS: 2,2-azino-bis (3-ethylbenzothiazoline-6-sulfonic acid)
AKI: acute kidney injury
AUC: area under the curve
BUN: blood urea nitrogen
DLS: dynamic light scattering
DMEM: Dulbecco's modified Eagle's medium
DMSO: dimethyl sulfoxide
DSF: differential scanning fluorimetry
FCS: fetal calf serum
GAPDH: glyceraldehyde-3-phosphate dehydrogenase
Hb: hemoglobin
HO-1: heme oxygenase 1
HO-1: heme oxygenase-1
HPLC: high-pressure liquid chromatography
i.m.: intramuscular
IPTG: isopropyl thiogalactoside
i.v.: intravenous
LDH: lactate dehydrogenase
ORAC: oxygen radical antioxidant capacity
PBS: phosphate-buffered saline
rA1M: recombinant human A1M
RIA: radioimmunoassay
ROS: reactive oxygen species
RT: room temperature
SDS-PAGE: sodium dodecyl sulfate/polyacrylamide gel electrophoresis
SEC: size exclusion chromatography
SPR: surface plasmon resonance
TFA: trifluoroacetic acid
wt: wild-type
