# Supplementary material for: rA1M-035, a Physicochemically Improved Human Recombinant α1-Microglobulin, Has Therapeutic Effects in Rhabdomyolysis-Induced Acute Kidney Injury
Source: Antioxid Redox Signal. 2018 Dec 27;30(4):489–504. doi: 10.1089/ars.2017.7181 (PMC6338582; doi:10.1089/ars.2017.7181)
Supplement: Supplemental data [file Supp_Fig1.pdf]

Supplementary Data

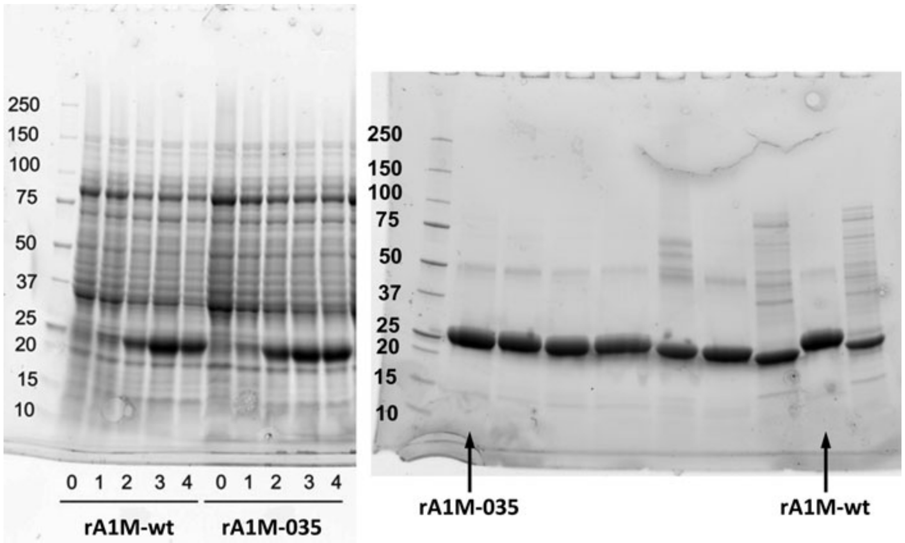

SUPPLEMENTARY FIG. S1. ***Left*****: Uncropped SDS-PAGE (4–20% gradient gel) of equal amounts of bacterial lysate from *E. coli* cultures expressing rA1M-wt and rA1M-035, taken from uninduced cultures (0), and 1, 2, 3, and 4 h after induction with IPTG**. *Right*: Uncropped SDS-PAGE (4–20% gradient gel) showing 10 μg of purified rA1M of various variants. rA1M-035 and rA1M-wt are marked. Both gels were stained with Coomassie. *Top* shows the application pockets and *bottom* shows the *bottom* end of the gels. Migration of size marker proteins is shown to the *left* of both gels. Cropped variants of the gels are shown in Figure 2 of the main article. A1M, α1-microglobulin; IPTG, isopropyl thiogalactoside; rA1M, recombinant human A1M; SDS-PAGE, sodium dodecyl sulfate/polyacrylamide gel electrophoresis; wt, wild-type.
