# Supplementary material for: rA1M-035, a Physicochemically Improved Human Recombinant α1-Microglobulin, Has Therapeutic Effects in Rhabdomyolysis-Induced Acute Kidney Injury
Source: Antioxid Redox Signal. 2018 Dec 27;30(4):489–504. doi: 10.1089/ars.2017.7181 (PMC6338582; doi:10.1089/ars.2017.7181)
Supplement: Supplemental data [file Supp_Fig2.pdf]

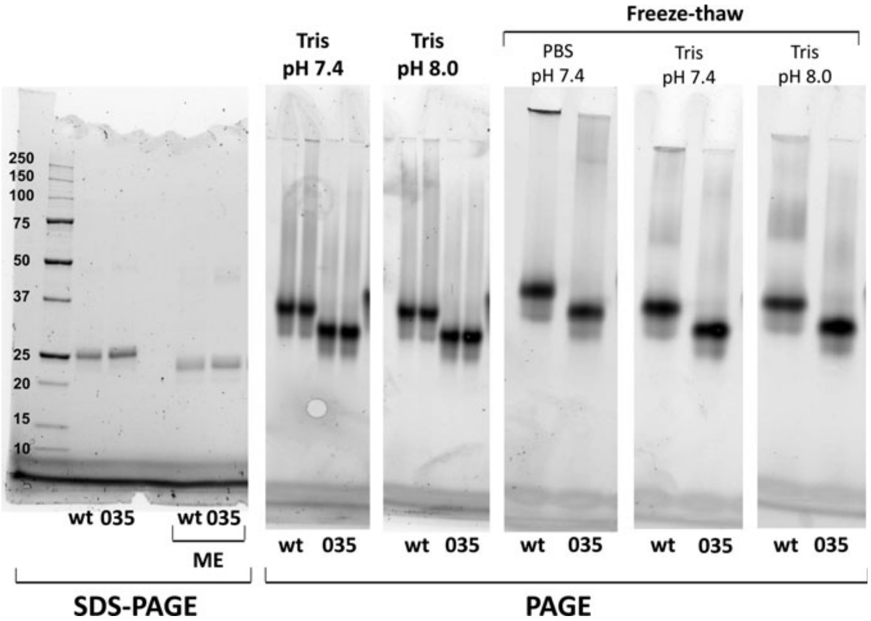

SUPPLEMENTARY FIG. S2. ***Left*: Uncropped SDS-PAGE (12% gel) of 0.8μg rA1M-wt and rA1M-035 under nonreducing and reducing mercaptoethanol (ME) conditions.** Migration of size marker proteins is shown to the *left* of the gel stained with Coomassie. A cropped variant of the gel is shown in Figure 3A of the main article. *Right:* Uncropped native PAGE (12% gel) of rA1M-wt and rA1M-035 treated under stress conditions as described in the Figure 3 legend in the main article. The gel was stained with Coomassie. *Top* shows the application pockets and *bottom* shows the *bottom* end of the gels. No size marker proteins were included since migration is based on both size and charge. Cropped variants of the gels are shown in Figure 3C of the main article.
