# Supplementary material for: rA1M-035, a Physicochemically Improved Human Recombinant α1-Microglobulin, Has Therapeutic Effects in Rhabdomyolysis-Induced Acute Kidney Injury
Source: Antioxid Redox Signal. 2018 Dec 27;30(4):489–504. doi: 10.1089/ars.2017.7181 (PMC6338582; doi:10.1089/ars.2017.7181)
Supplement: Supplemental data [file Supp_Fig3.pdf]

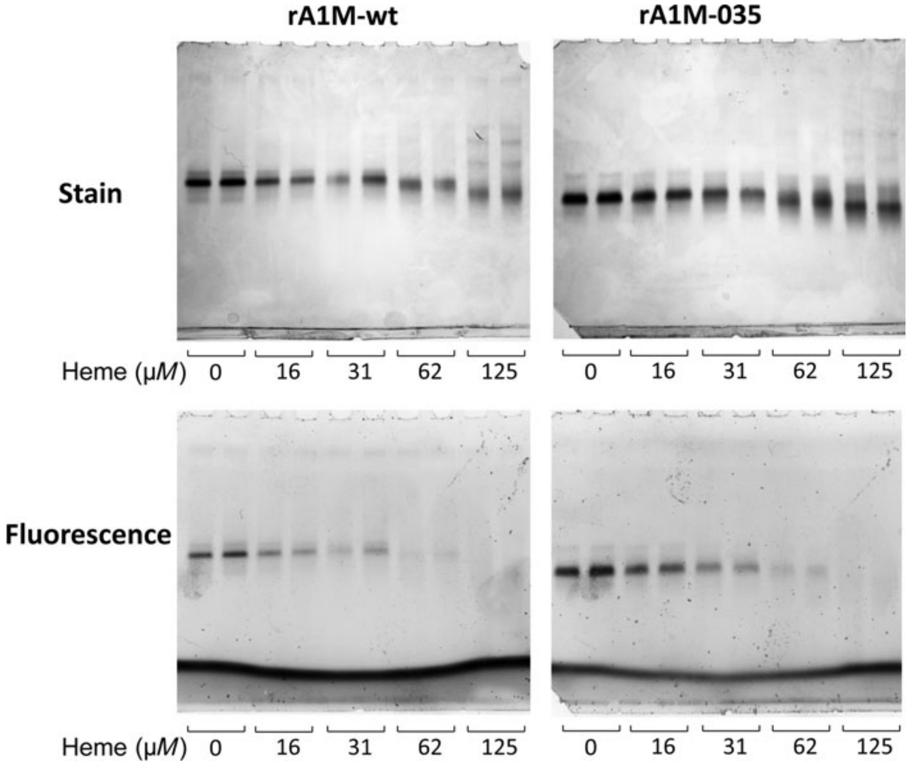

SUPPLEMENTARY FIG. S3. Uncropped native PAGE of 15 μg rA1M-wt or rA1M-035 incubated with different amounts of heme. The gels were analyzed by densitometry scanning after Coomassie staining (stain) and tryptophan fluorescence (fluorescence) as described in the Figure 5A legend in the main article. *Top* shows the application pockets and *bottom* shows the *bottom end* of the gels. No size marker proteins were included since migration is based on both size and charge. Cropped variants of the gels are shown in Figure 3C of the main article.
